# A Review of Yarn-Based One-Dimensional Supercapacitors

**DOI:** 10.3390/nano13182581

**Published:** 2023-09-18

**Authors:** Duri Han, Minju Kim, Sojung Lee, Changsoon Choi

**Affiliations:** Department of Energy and Materials Engineering, Dongguk University, 30 Pildong-ro, 1-gil, Jung-gu, Seoul 04620, Republic of Korea; gksenfl1234@dgu.ac.kr (D.H.); dudhkdalswn@dgu.ac.kr (M.K.); sojung010104@gmail.com (S.L.)

**Keywords:** 1D energy storage, yarn supercapacitors, wearable devices, flexibility, stretchability

## Abstract

Energy storage in a one-dimensional format is increasingly vital for the functionality of wearable technologies and is garnering attention from various sectors, such as smart apparel, the Internet of Things, e-vehicles, and robotics. Yarn-based supercapacitors are a particularly compelling solution for wearable energy reserves owing to their high power densities and adaptability to the human form. Furthermore, these supercapacitors can be seamlessly integrated into textile fabrics for practical utility across various types of clothing. The present review highlights the most recent innovations and research directions related to yarn-based supercapacitors. Initially, we explore different types of electrodes and active materials, ranging from carbon-based nanomaterials to metal oxides and conductive polymers, that are being used to optimize electrochemical capacitance. Subsequently, we survey different methodologies for loading these active materials onto yarn electrodes and summarize innovations in stretchable yarn designs, such as coiling and buckling. Finally, we outline a few pressing research challenges and future research directions in this field.

## 1. Introduction

**Figure 1:**
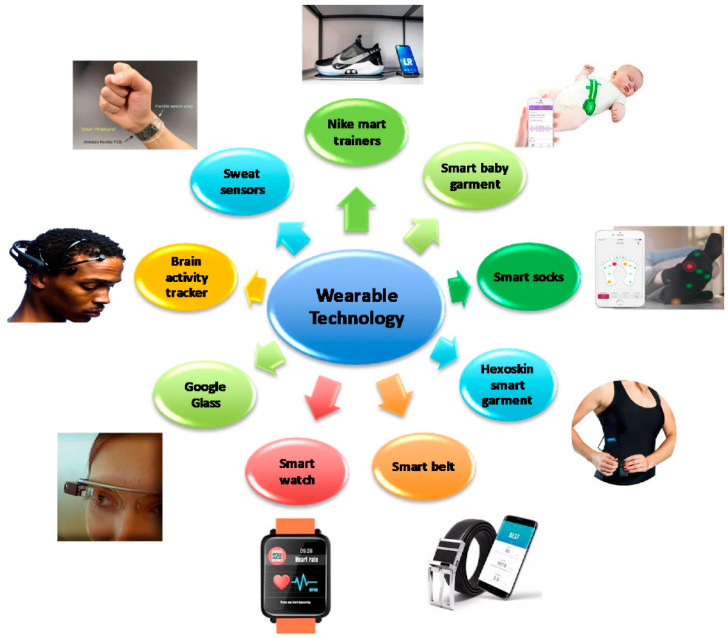
Various examples of wearable technologies. Reproduced under the terms of the CC BY 4.0 license [8].

**Figure 2:**
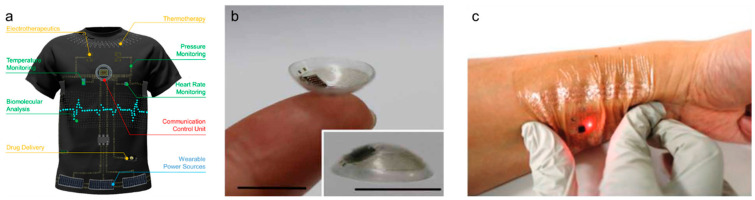
(**a**). Representative functional units of intelligent wearable point-of-care textile systems, involving diagnostic, therapeutic, and protective devices, as well as wearable power sources. Reprinted with permission [9]. Copyright 2022, American Chemical Society. (**b**). Photographs of a smart contact lens (inset: close-up outer image of the smart contact lens) (scale bars = 1 cm). Reproduced under the terms of the CC BY-NC 4.0 license [10]. (**c**). Photograph of a wireless electronic tattoo under compressive deformation and worn on the skin. Reproduced with permission [11]. Copyright 2019, Wiley-VCH.

The advent of the Fourth Industrial Revolution has initiated a new phase of hyper-personalization and hyper-connectivity in which individual electronics are linked (Figure 1) [1]. Furthermore, leading global technology companies are unveiling an array of prototypes pertaining to wearable sensors and displays [2]; however, research on wearable energy storage systems is presently limited [3]. To reliably supply energy to next-generation flexible and deformable wearable devices, developing next-generation energy-storage media that are compatible with these advanced technologies is a pressing requirement. Yarn-based one-dimensional (1D) supercapacitors, an ensemble of smart-textile technology, are the optimal choice for wearable energy storage devices for several reasons [4]. Yarn or fiber structures, with diameters ranging from tens to hundreds of micrometers and lengths spanning the range from centimeters to meters, offer a unique set of advantages, including flexibility, light weight, small form factor, and high scalability. Furthermore, supercapacitors offer distinct advantages over other types of energy storage devices (e.g., batteries) when used in wearable energy sources. First, these supercapacitors offer faster charge/discharge cycles and higher power densities than those offered by batteries owing to their unique charge storage mechanisms [5]. Second, supercapacitors have extended lifespans owing to their broad operating temperature range and excellent cycle stability [6]. Third, supercapacitors contain environmentally friendly aqueous electrolytes, unlike conventional batteries, which may contain hazardous metals and electrolytes. Lastly, supercapacitors are fabricated using relatively simple components, making these supercapacitors ideal for integration into yarn structures. These benefits allow for a wide range of applications in various sectors, such as smart clothing, healthcare, and the military (Figure 2) [7]. This paper offers an overview of the existing literature on and trends in yarn-based supercapacitors. Initially, we explore the unique characteristics and operational mechanisms of supercapacitors. Next, we provide a summary of recent advancements categorized according to the materials, fabrication techniques, and electrode structures. We then conclude by highlighting the current technical challenges and future directions in this field of research.

## 2. Basics of Supercapacitors

### 2.1. Types of Supercapacitors

**Figure 3:**
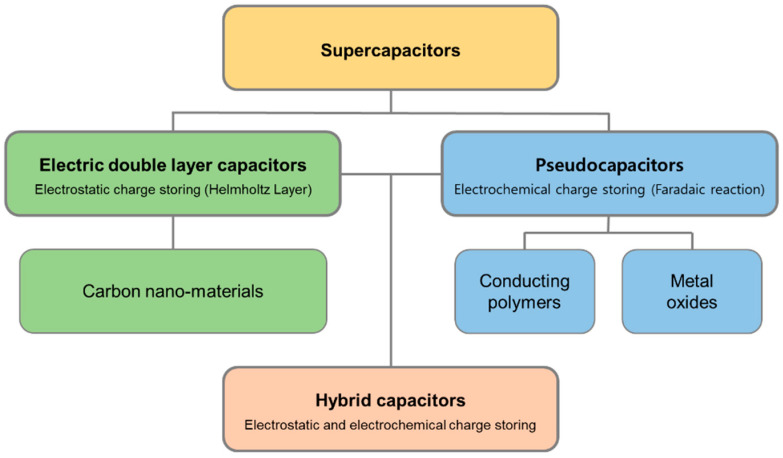
Supercapacitor types: electric double-layer capacitors, pseudocapacitors, and hybrid capacitors, as defined by their designs.

Supercapacitors, also known as electrochemical capacitors or ultracapacitors, are energy storage systems that store electrical power through either physical ion adsorption/desorption or localized electrochemical processes occurring at the electrode–electrolyte interface [12,13,14]. Supercapacitors also share some basic components with batteries, namely, (1) two electrodes at which electrochemical reactions occur, (2) an electrolyte that serves as an ion-conducting medium, and (3) a separator to avoid physical contact between the two electrodes. Supercapacitors offer faster charge/discharge rates, higher power outputs, and considerably better cycle lives than those offered by traditional batteries. Based on the type of active material used for charge storage, supercapacitors can be categorized into three primary types: (1) electric double-layer capacitors (EDLCs), (2) pseudocapacitors, and (3) hybrid capacitors (Figure 3). Each type employs a distinct charge storage mechanism.

### 2.2. Working Mechanism

**Figure 4:**
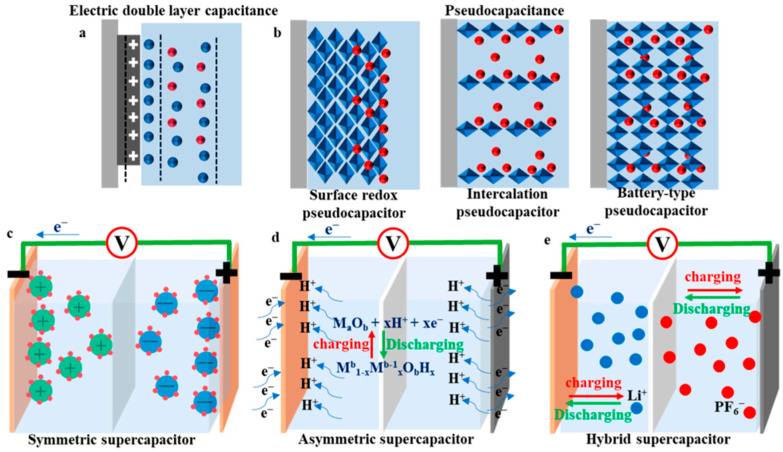
Schematics showing the operating mechanisms of (**a**) an EDLC, (**b**) a pseudocapacitor, (**c**) a symmetric supercapacitor, (**d**) an asymmetric supercapacitor, and (**e**) a hybrid supercapacitor. Reproduced under the terms of the CC BY 4.0 license [20].

EDLCs store electrical energy by forming an electric double layer at the interface between the electrodes and the electrolyte. When electrodes are subjected to a voltage within an electrolyte, ions are physically adsorbed onto the electrode surface owing to electrostatic forces. As illustrated in Figure 4a, these ions are separated from the electrode by an inner Helmholtz plane, leading to the formation of an electric double layer [15,16]. This arrangement resembles that of a traditional capacitor that consists of two parallel plates separated by a dielectric. The capacitance (*C*), representing the charge storage capacity, is calculated using the ratio of the stored charge (*Q*) to the applied voltage (*V*), i.e., *C* = *Q*/*V*. The capacitance is directly related to the dielectric constant, *ε*, and the surface area of the electrode, *A*, as well as inversely related to the electrode separation distance, *d* (*C* = *εA*/*d*). Owing to the large surface area of the electrode and the short distance between the layers, which is nearly on the Angstrom scale (1 Å = 10^−10^ m), EDLCs can achieve much higher energy densities than those achieved by traditional capacitors. By contrast, pseudocapacitors store charge through Faradaic reactions at the electrode–electrolyte interface. This charge storage mechanism involves ion movements in the electrolyte, redox reactions at the electrode surface, and electron transfer events at the electrode–electrolyte interface, as depicted in Figure 4b. The redox reactions in batteries are confined to the specific potentials of the active materials. However, when nanosized materials are evenly dispersed on electrode surfaces, continuous energy bands emerge from the overlapping electron orbits of adjacent atoms. This allows for redox reactions across a broad range of voltages via continuous electron transfer to each energy state [17]. This phenomenon is termed pseudocapacitance and results in capacitances that are 5–100 times higher than the capacitance of EDLCs for an identical electrode surface. Conway first identified this effect during the 1970s while examining RuO_2_; presently, this effect is noticeable in a range of metal oxides, including MnO_2_, nitrides, and carbides [18,19].

Hybrid capacitors combine the traits of both capacitors and batteries by using asymmetrical electrodes, one of which features a capacitor-like high-power material, while the other is made of a battery-like high-capacity material, as displayed in Figure 4d,e [14,19]. Although these capacitors function under a large operating voltage range and enhanced energy density, they pose challenges owing to their nonlinear charge/discharge profiles and control complexities with respect to practical applications.

### 2.3. Comparisons

**Figure 5:**
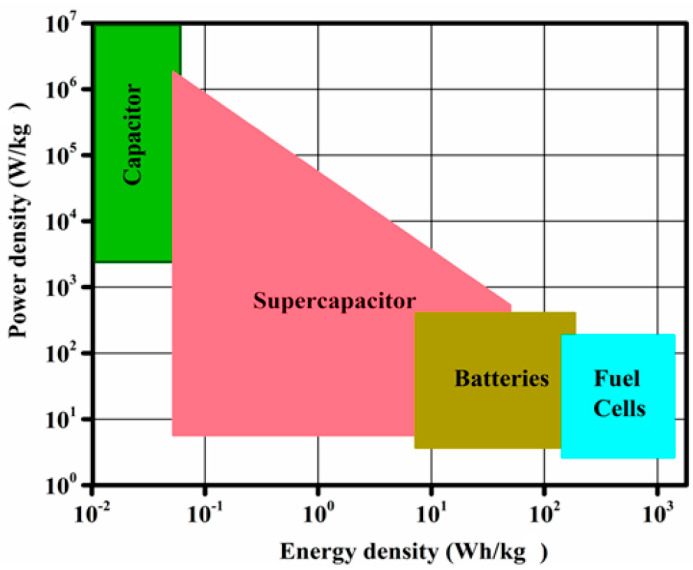
Ragone graph showing the performance of the major energy storage devices, including batteries, capacitors, and electrochemical supercapacitors, in terms of the energy and power densities. Reproduced under the terms of the CC BY 4.0 license [21].

Figure 5 shows a Ragone plot comparing the energy and power densities of different types of energy storage devices. The graph shows that supercapacitors are present in a region between conventional capacitors and batteries, revealing that supercapacitors offer higher energy and power densities than conventional capacitors and batteries, respectively [21]. These distinctions arise from the unique charge storage mechanism inherent to each type of device. Batteries rely on redox reactions to store significant amounts of electrical energy, although they exhibit long charge/discharge cycles, low power densities, and limited cycle lives. Conversely, supercapacitors benefit from localized interactions at the electrode–electrolyte interface, which yield rapid charge/discharge times, high power densities, and notably durable cyclic performances [18]. Furthermore, batteries contain heavy and voluminous active materials and intricate components and require complex fabrication procedures; therefore, batteries are mostly unsuitable for applications in flexible and stretchable yarn structures. Batteries also present challenges related to their energy efficiency and environmental impacts, particularly in relation to wearable applications [14]. In contrast, supercapacitors employ nanoscale active materials, use environmentally friendly and nonflammable electrolytes, and contain components with simple structures. Therefore, supercapacitors are increasingly being considered promising next-generation alternatives to traditional batteries for wearable energy storage applications [14,19].

## 3. Research Trends concerning Yarn Supercapacitors

### 3.1. Materials

Yarn electrodes serve as the foundation for the development of yarn-based supercapacitors. These sub-electrodes need to simultaneously function as current collectors for electron transport, substrates for maintaining the yarn structure, and occasionally, as energy storage materials. Among several candidates, carbon nanomaterials, such as carbon nanotubes (CNTs) and graphene, are considered the most suitable electrode materials for yarn-based supercapacitors, while various materials, including metal oxides and conductive polymers, have been used as active materials. Although electrolytes are important components, the research available on them is relatively less compared with that on electrodes. In the following section, we introduce recent research trends concerning carbon-nanomaterial-based yarn supercapacitors with metal oxides and conductive polymers as their active materials.

#### 3.1.1. Carbon Nanomaterials

**Figure 6:**
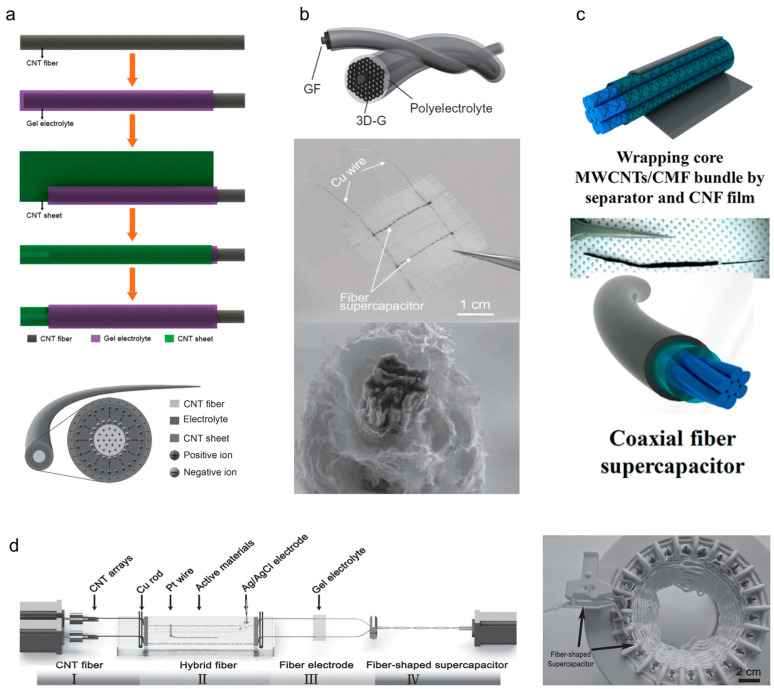
(**a**) Schematics showing the fabrication of coaxial EDLC fiber bases on a CNT fiber and sheet as two electrodes and the cross-sectional structure of the coaxial EDLC fiber. Reproduced with permission [22]. Copyright 2013, Wiley-VCH. (**b**) Schematic of a wire-shaped supercapacitor fabricated from two twined graphene fibers and a three-dimensional porous graphene framework with polyelectrolyte, an optical photograph of a textile embedded with two fiber supercapacitors, and a cross-sectional scanning electron microscope image showing the core graphene fiber surrounded by standing graphene sheets. Reproduced with permission [23]. Copyright 2013, Wiley-VCH. (**c**) Schematics and digital photographs showing the fabrication procedures for a coaxial fiber supercapacitor. After soaking in polymer electrolyte, the core MWCNTs/CMF bundle was wrapped in a separator and CNF film. Reprinted with permission [24]. Copyright 2013, American Chemical Society. (**d**) Schematic of the experimental setup used for the continuous fabrication of the supercapacitor fiber and photograph of the knitting process (the arrow shows the supercapacitor fiber). Reproduced with permission [27]. Copyright 2015, Wiley-VCH.

Scanning carbon nanomaterials (e.g., CNTs, graphene, and carbon fibers) have been widely used as electrode materials in yarn supercapacitors owing to the excellent electrical conductivity, flexibility, large surface area, light weight, and chemical stability of these nanomaterials. Carbon-based yarn supercapacitors are generally referred to as electrochemical double-layer capacitors, as their electrical charges are stored electrostatically on the surfaces of the carbon electrodes owing to physical adsorption, which is also called the electrical double-layer effect. Yarn electrodes containing carbon nanomaterials are primarily manufactured via large-scale production techniques such as dry spinning, wet spinning, or hydrothermal assembly. The carbon-based active materials, typically CNT and reduced graphene oxide (rGO) in the form of particles or flakes, are loaded onto the electrodes via the spray coating, dip coating, or electrochemical deposition methods. Peng and colleagues have pioneered and researched carbon-nanomaterial-based yarn supercapacitors. Figure 6a presents the schematics of the fabrication procedures and cross-sections of CNT yarn and CNT sheet-based yarn supercapacitors [22]. Here, the CNT fibers are coated with a gel-type polyvinyl alcohol (PVA)/H_3_PO_4_ electrolyte and subsequently wrapped with CNT sheets. Finally, the fiber devices are coated with the same gel electrolyte again to allow for the infiltration of the electrolyte into the outermost CNT layer such that the resulting fiber yields a high specific capacitance of 50 F/g. Graphene is also a representative electrode material for yarn-type supercapacitors. Meng et al. [23] reported a graphene-based fiber-type supercapacitor in which a graphene core fiber was wrapped in a porous graphene network (Figure 6b). As a graphene network has high electrical conductivity and a large surface area, the resulting supercapacitor delivers outstanding energy and power densities of 0.17 μWh/cm^2^ and 100 μW/cm^2^, respectively. As shown in Figure 6c, Le et al. [24] reported carbon fiber and multiwalled nanotube (MWNT)-based yarn supercapacitors. Here, the fiber electrode consists of MWNT/carbon microfiber bundle composites as the center and a carbon nanofiber film produced via electrospinning as the outer layer to form a coaxial structure. As a result, the coaxial fiber supercapacitor exhibits a capacitance of 86.8 mF/cm^2^. In addition, a graphene oxide (GO) fiber supercapacitor that contains a small amount of rGO produced by reducing GO using a laser has been reported [25]. The GO separated the rGO to prevent physical contact (separator), while the rGO stored ions by forming an electric double layer and providing an electron transport pathway (active material and current collector). Furthermore, yarn electrodes that can be continuously fabricated are essential for practical applications of yarn-based supercapacitors. Wet spinning is a well-known method for the mass production of CNT and graphene fibers. Chen et al. [26] manufactured several meter-long GO fibers via the conventional wet spinning of a GO dispersion; subsequently, a chemical treatment with an acidic solution was used to reduce the graphene, resulting in the production of rGO-based graphene fibers with high electrical conductivity and excellent mechanical stability. Figure 6d shows the continuous fabrication procedure applied to obtain rGO/CNT composite-fiber-based supercapacitors [27]. The aligned CNT fibers drawn from the CNT array were immersed in a GO dispersion solution such that the GO was deposited and reduced via the electrochemical treatment, resulting in rGO and CNT composite-based fiber supercapacitors. This continuously fabricated fiber supercapacitor can be scaled up by weaving and knitting it with commercially available fibers or fabrics.

#### 3.1.2. Metal Oxides

**Figure 7:**
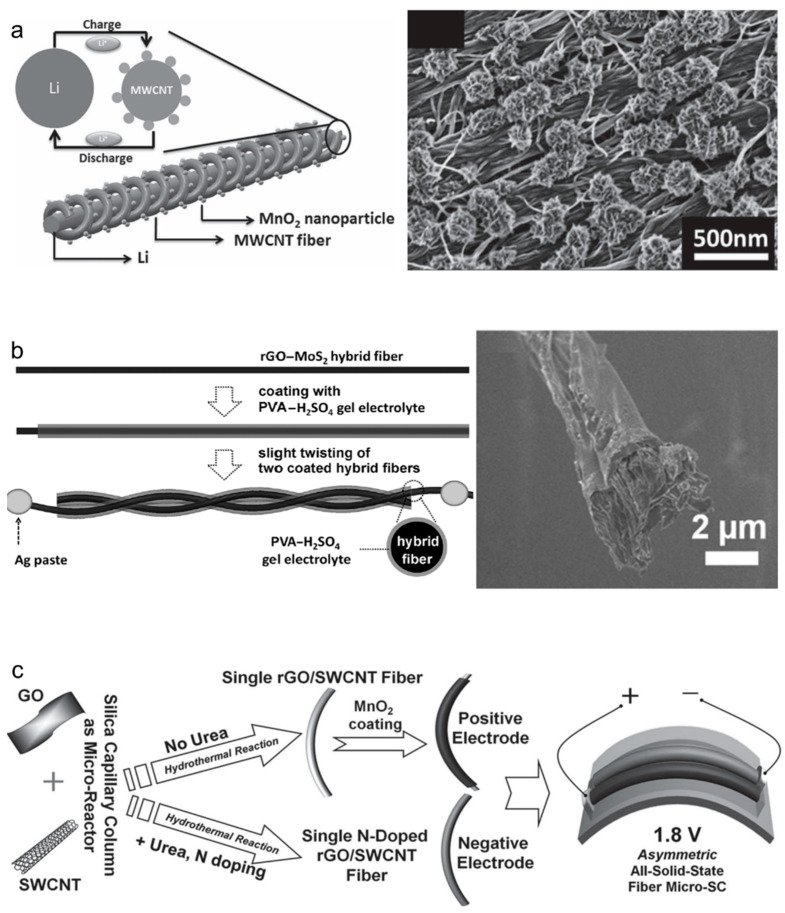
(**a**) Schematic showing the wire-shaped lithium-ion battery fabricated by twisting an aligned MWCNT/MnO_2_ composite fiber and lithium wires as the positive and negative electrodes. A scanning electron microscope (SEM) image showing the aligned MWCNT fibers after the electrodeposition of MnO_2_ nanoparticles. Reproduced with permission [28]. Copyright 2012, Wiley-VCH. (**b**) Schematic showing the fabrication procedure for and cross-sectional SEM image of an rGO-MoS_2_ hybrid fiber coated with a PVA-H_2_SO_4_ gel electrolyte. Reproduced with permission [29]. Copyright 2014, Wiley-VCH. (**c**) Schematics showing the materials and fabrication procedures related to an asymmetric all-solid-state fiber-based microsupercapacitor. Reproduced with permission [31]. Copyright 2014, Wiley-VCH.

Metal oxides can be used to prepare high-performance yarn-based supercapacitors. When nanosized metal oxides are closely distributed on an electrode’s surface, the overlapping electron orbits form a continuous energy band, resulting in the pseudocapacitance effect by which continuous electron transfer occurs at each energy state over a wide voltage range. Supercapacitors formed using pseudocapacitive materials are called pseudocapacitors, and their charge storage performances (typically areal capacitances) are up to 100 times greater than those of EDLCs. Since Conway discovered pseudocapacitance in RuO_2_ in 1970, these effects have been observed in diverse materials, such as nitrides, carbides, and metal oxides. To maintain the nano-scale dimensions, pseudocapacitive materials are typically loaded onto prepared carbon-based yarn electrodes via electrochemical deposition. Otherwise, the bi-scrolling method, which involves drop-casting a solution containing metal oxide nanoparticles onto sheet-like carbon electrodes and scrolling the sheets into yarns, is also widely employed. The detailed fabrication procedures are introduced in the following section. Currently, MnO_2_, which is inexpensive, harmless, and easy to produce as nanoparticles, is the most widely used active material in pseudocapacitors. However, the poor electrical conductivities and coulombic efficiencies of metal oxides are major issues that need to be overcome via future research. Ren et al. [28] reported a wire-type supercapacitor made using MWNT and MnO_2_. The MnO_2_ was electrochemically deposited on an MWNT core fiber, which was fabricated by inserting twists into an aligned CNT sheet (Figure 7a). The amount of deposited MnO_2_ was controlled by controlling the deposition time (the maximum weight% of MnO_2_ was approximately 4.1). The electrochemical capacitance increased as the MnO_2_ deposition time increased, yielding final areal and specific capacitances of 3.01 mF/cm^2^ and 13.31 F/g, respectively. Sun et al. [29] used MoS_2_, a transition-metal dichalcogenide, as the active material. The electrode was fabricated by wet spinning a GO and MoS_2_ mixture, and the GO in the spun-fiber electrode was chemically reduced to rGO (Figure 7b). The volumetric capacitance of the rGO-MoS_2_ fiber supercapacitor was 10 times higher (30 F/cm^3^ at 2.2 wt% of MoS_2_) than that of the rGO fiber supercapacitor. Ma et al. [30] used rGO and MnO_2_ to fabricate composite-fiber supercapacitors by wet spinning GO synthesized via the Hummers method and MnO_2_ nanowires produced using a low-temperature hydrothermal method, followed by the chemical reduction of GO. The electrochemical capacitance was optimized by adjusting the mass ratios of the GO and MnO_2_ in the mixture solution, and a high volumetric capacitance was achieved (66.1 F/cm^3^ at 40% of MnO_2_). Furthermore, an asymmetric electrode-based fiber supercapacitor that uses different materials at the anode and cathode has been reported [31]. As shown in Figure 7c, each electrode was prepared via the MnO_2_ coating and N-doping of GO and single-walled nanotube (SWNT)-based composites, respectively. The asymmetric electrodes operated under wide voltage windows of up to 1.8 V, resulting in an increased linear capacitance of 3.3 mF/cm.

#### 3.1.3. Conductive Polymers

**Figure 8:**
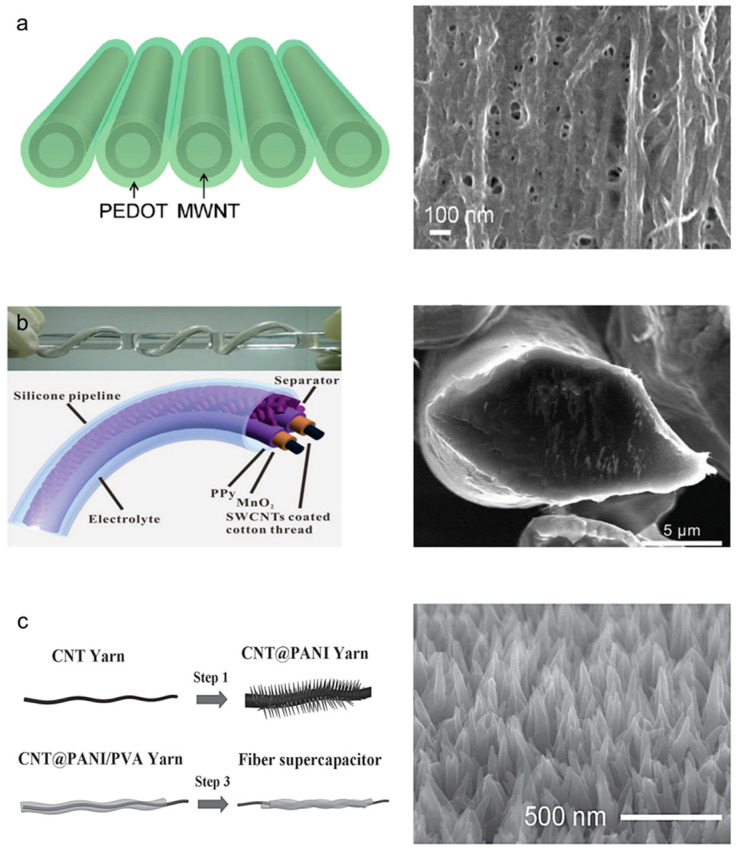
(**a**) Schematic and SEM image showing a PEDOT-coated CNS nanomembrane. Reprinted with permission [32]. Copyright 2012, American Chemical Society. (**b**) Schematic and optical photograph showing a twisting-cable-type fiber supercapacitor coated with Ppy, and cross-sectional SEM images of an SWCNT-coated microfibril. Reproduced with permission [34]. Copyright 2013, Wiley-VCH. (**c**) Schematics showing the fabrication procedures for and SEM image showing the magnified surface of a PANI-coated CNT composite yarn. Ordered PANI nanowire arrays can be observed in the SEM image. Reproduced with permission [35]. Copyright 2013, Wiley-VCH.

Conductive polymers are another representative pseudocapacitive material. Certain polymers containing alternating single and double bonds in a conjugated structure exhibit pseudocapacitive effects owing to the presence of delocalized electrons and continuous electron transfer. Currently, poly(3,4-ethylenedioxythiophene) (PEDOT) is actively used as a pseudocapacitive material for yarn-based supercapacitors owing to its electrical conductivity, mechanical flexibility, and cost-effectiveness. In addition, various conductive polymers, such as polypyrrole (Ppy) and polyaniline (PANI), are also widely applied. However, their relatively poor capacitances and cyclic performances remain challenges. The most prevalent techniques for introducing conductive polymers into yarn electrodes include various polymerization (vapor phase or in situ), bi-scrolling, and electrochemical deposition methods. Lee et al. [32] reported PEDOT-coated MWNT yarn-based supercapacitors. Figure 8a shows the schematic and a scanning electron microscope (SEM) image of aligned CNT bundles coated with gas-phase-polymerized PEDOT. MWNT yarn-based supercapacitors with 85 wt% PEDOT exhibited enhanced volumetric energy and power density of 47 mWh/cm^3^ and 538 W/cm^3^, respectively. Furthermore, a volumetric capacitance of 179 F/cm^3^ was achieved when the bi-scrolling technique was applied, whereby the active material was contained within a fiber electrode, in a follow-up study [33]. Liu et al. [34] fabricated a cable-type supercapacitor in which SWNTs and MnO_2_ were loaded on conventional porous cotton and wrapped with Ppy (Figure 8b). The outstanding electrical conductivity of Ppy effectively overcame the low electrical conductivity of MnO_2_, and Ppy exhibited an excellent specific capacitance of 1370 F/g. Additionally, the possibilities of commercialization and mass production were demonstrated using 2 m long fiber electrodes prepared from commercially available cotton fibers. Furthermore, PEDOT, Ppy, and PANI are widely used as conductive-polymer active materials. As shown in Figure 8c, PANI is deposited on the twist-inserted CNT yarn, resulting in a 16-fold increase in the areal capacity (38 mF/cm^2^) with respect to that of a pristine CNT yarn [35]. Zhu et al. [36] deposited gold nanoparticles and PANI onto CNT yarn electrodes, showing improved capacitances of more than five times (8.7 F/g). Cao et al. [37] reported PANI-based coaxial yarn-based supercapacitors that were fabricated via electrochemical PANI deposition on CNT yarn for the electrode and PANI nanofiber electrospinning for the interlayer. The PANI layer with a thickness of approximately 1 μm physically separated both electrodes and prevented an electrical short circuit. As a result, an areal capacitance of 560 mF/cm^2^ was achieved to operate 10 light-emitting diodes (LEDs) using serial and parallel connections of PANI yarn supercapacitors. During practical use, no significant performance degradation was observed under bending deformation, demonstrating the suitability of these supercapacitors for wearable applications. The electrochemical energy storage performances of various active materials are listed in Table 1.

### 3.2. Structures

In the context of current technological advancements, the electrochemical energy density of yarn supercapacitors must be increased to allow for the manufacturing of effective power electronic devices. The primary research focus has been on material science approaches, specifically on electrode materials that offer large specific surface areas or exhibit pseudocapacitance effects. However, given the inherent spatial constraints of a 1D yarn structure, which offers limited surface area and volume, yarn-electrode architectures and effective methods must be developed for loading active materials by optimizing the use of the available material-loading sites. The operational stability of yarn-based supercapacitors is another consideration, particularly owing to the diverse mechanical and chemical stresses that they may encounter during real-world applications. This underscores the importance of developing groundbreaking electrode structures and loading techniques for active materials. The subsequent sections of this paper discuss the current research trends aimed at improving both the energy-storage performances and operational stabilities of these devices through advancements in electrode architectures and loading methods for active materials.

#### 3.2.1. Core–Shell Structures

**Figure 9:**
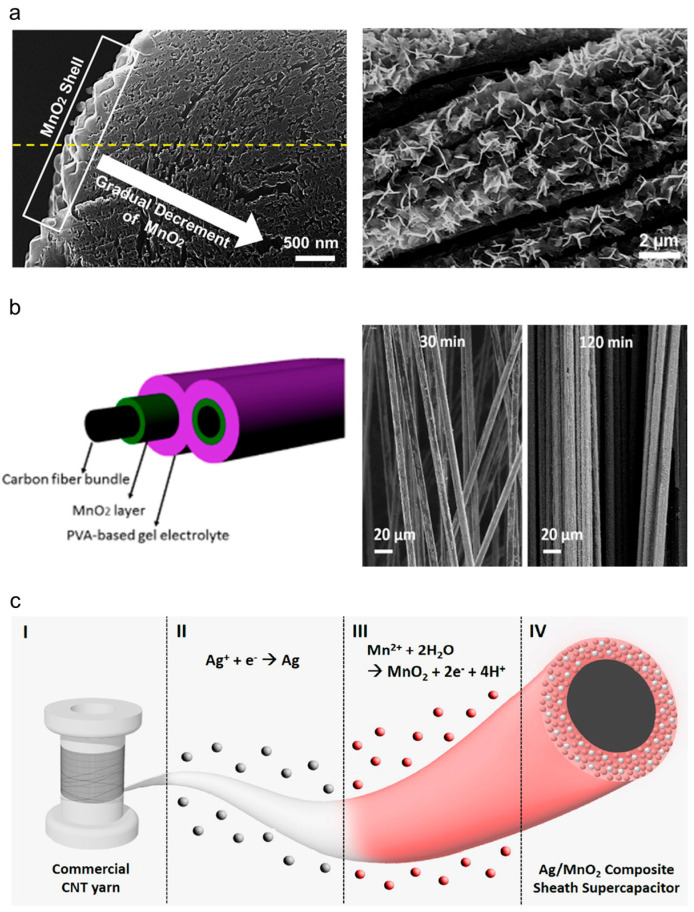
(**a**) Magnified SEM images showing the edges and the surface of a core–shell-structured CNT/MnO_2_ yarn-based supercapacitor. Well-coated flower-shaped MnO_2_ particles can be observed. Reproduced with permission [39]. Copyright 2013, Wiley-VCH. (**b**) Schematic showing the cross-section of an MnO_2_-coated carbon fiber supercapacitor, and SEM images showing the surfaces of carbon fibers with different MnO_2_ deposition times. Reprinted with permission [40]. Copyright 2017, American Chemical Society. (**c**) Schematic showing the fabrication procedure for an Ag/MnO_2_ composite sheath yarn, (I) commercial CNT yarn, (II) Ag electrodeposition, (III) MnO_2_ electrodeposition, and (IV) Ag/MnO_2_ composite sheath yarn supercapacitor. Reproduced under the terms of the CC BY 4.0 license [42].

The structure of a yarn supercapacitor electrode is determined by the sequential relationship between the yarn fabrication and the steps for loading the active material. The core–shell structure is a concentric structure formed by loading an active material after fabricating the yarn electrode. The conductive yarn-based center (core) functions as a current collector, whereas the energy-storing active materials constitute the outer layer (shell). Common methods for manufacturing core–shell structures include electrochemical deposition, dipping, drop casting, and spraying. Such structures are relatively easy to realize by controlling the weights of the loaded active material, which makes this process advantageous for mass production, leading to active research and reports on the early stages of mass-producing yarn-based supercapacitors. However, only the shell, which is a part of the entire yarn, participates in electrochemical reactions, resulting in reduced energy storage performance. Therefore, increasing the weight of the loaded active material to overcome this limitation leads to the formation of a thick shell. The metal oxides comprising the shell have poor electrical conductivity and extend the electron-transport pathway, thereby increasing the internal resistance and overvoltage, which degrades the performances of the supercapacitors. Furthermore, the brittleness of the metal oxide deteriorates the mechanical properties and flexibility of yarn electrodes, leading to the detachment of the active material under external forces. To address this issue, a porous material with a large specific surface area is applied to the core current collector, while conductive additives are introduced into the active-material shell to reduce internal resistance. Chen et al. [38] reported fiber supercapacitors that were fabricated by depositing MnO_2_ onto hierarchical graphene fibers. After deposition, a thick shell of MnO_2_ was formed on the surface of the fiber, showing linear and specific capacitances of 0.143 mF/cm and 36 F/g, respectively. Choi et al. [39] fabricated a core–shell-structured yarn-based supercapacitor via the electrochemical deposition of MnO_2_ onto a CNT yarn (Figure 9a). The MnO_2_ shell was gradually distributed from the outermost layer to the inside of the electrode owing to the nanobundles and numerous pores of the CNT yarn. Therefore, an excellent volumetric capacitance of 25.4 F/cm^3^ and approximately 10 times the capacitance retention performance were observed during rapid charging and discharging with respect to those of conventional core–shell yarn-based supercapacitors (28.3% at 3 V/s). Rafique et al. [40] loaded MnO_2_ onto carbon fibers via electrochemical deposition for up to 2 h. Depending on the deposition time, different amounts of MnO_2_ were loaded on the carbon fibers (Figure 9b). The linear and specific capacitances obtained were 23 mF/cm and 62 F/g, respectively. Wang et al. [27] demonstrated the possibility of the mass production of yarn supercapacitors by reducing the entire fabrication procedure to less than 5 min by electrochemically depositing various pseudocapacitive materials (e.g., rGO, MnO_2_, PANI, and Ppy). The resulting MnO_2_-deposited yarn-based supercapacitors exhibited a volumetric capacitance of 20.2 F/cm^3^ and a specific capacitance of 34.9 F/g. Zhang et al. [41] optimized the amounts of MnO_2_ by adjusting the deposition times. Volumetric and specific capacitances of 58.7 F/cm^3^ and 428 F/g, respectively, were obtained with a deposition time of 500 s. Moreover, Kim et al. [42] mitigated the capacitance retention performance, which is caused by the presence of a thick layer of metal oxide, by introducing a conductive additive to the thick active-material layer (Figure 9c). MnO_2_ (active material) and Ag (conductive additive) were alternately deposited onto commercially available CNT yarn to form a mixed shell. As a result, an areal capacitance of 322 mF/cm^2^ and twice the retention performance were achieved with respect to those of the conventional core–shell structure during fast charging and discharging (42.6% at 100 mV/s).

#### 3.2.2. Embedded Structures

Despite the ease of fabrication, core–shell structures have severe restrictions, i.e., the core yarn rarely contributes to the energy storage capacitance and the loading weight of the active material is severely limited (under 20 wt%). To overcome the structural limitations, the shell and inner core must be incorporated into the loading site of the active material. Embedded structures are formed when the yarns are fabricated using active-material-loaded electrodes, utilizing all areas of the fiber electrodes as loading sites for the active material. This structure contains evenly distributed (or interlaminated) metal oxides in the gaps of the porous electrode, maximizing the active material content to more than 99 wt% (containing 99 times the weight of the current collector). In addition, the random distribution of the active material efficiently shortens the electron transport pathway, resulting in high capacitance and superior retention performances, while the physical fixation of the current collector prevents the active material from detaching under strong external forces or mechanical deformation. However, as precise control of the active material content is difficult and the brittleness of the active material degrades the mechanical properties and flexibility of the yarn electrode, the active material content must be optimized. The bi-scrolling fabrication method, which applies twists in the composite electrode mixed with the current collecting host material and energy storage active material guest, is mainly used to realize embedded structures.

**Figure 10:**
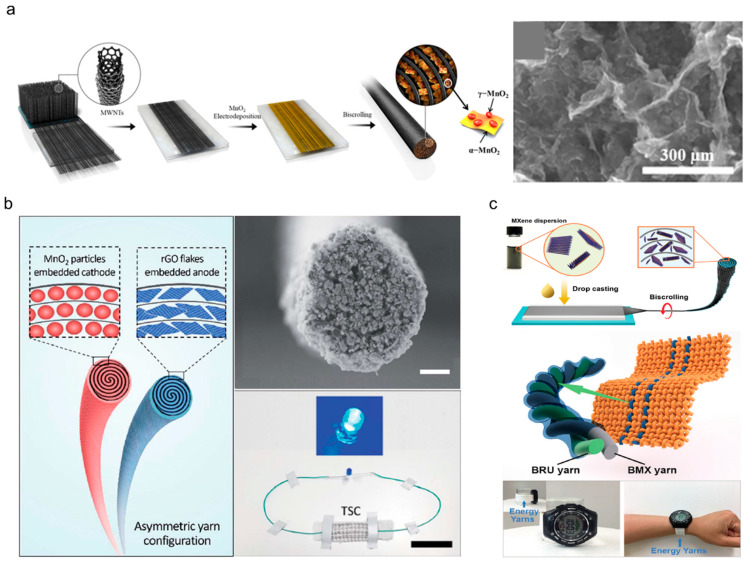
(**a**) Schematics showing the fabrication procedure for and SEM image showing the surface morphology of an MnO_2_-deposited CNT yarn-based supercapacitor. Reproduced under the terms of the CC BY 4.0 license [43]. (**b**) Schematics showing an asymmetrically configured yarn-based supercapacitor with an MnO_2_-embedded yarn cathode and rGO-embedded yarn anode. SEM image showing the cross-section of an MnO_2_-embedded yarn cathode, and optical photograph of a blue LED light powered by the asymmetrically configured yarn-based supercapacitor (scale bar = 15 μm and 4 cm). Reproduced under the terms of the CC BY 3.0 license [44]. (**c**) Schematics showing the fabrication procedures for MXene-embedded CNT composite yarn-based supercapacitors and an energy textile prototype containing yarn-based supercapacitors. Optical photographs of a digital watch powered by the energy textile prototype. Reproduced with permission [45]. Copyright 2018, Wiley-VCH.

Conductive polymers, nanosized metal-oxide particles, and carbon-based flakes are the most widely used guest materials in embedded yarn-based supercapacitors. The dispersion solutions of the guest materials are drop-cast onto sheet- or film-type carbon hosts. Subsequently, the guest-loaded host is scrolled into the yarn-structured electrode. Sun et al. [29] reported PEDOT-embedded CNT yarn-based supercapacitors in which PEDOT was coated on aligned CNT sheets with excellent electrical conductivity and mechanical properties. The sheets were then twisted to fabricate yarn electrodes. Enhanced energy and power densities (47 mWh/cm^3^ and 538 W/cm^3^, respectively) and outstanding retention performance were demonstrated even at a fast charge/discharge rate of 25 V/s. Figure 10a shows the fabrication procedures used for MnO_2_-embedded CNT yarn-based supercapacitors [43]. MnO_2_ nanoparticles were electrochemically deposited onto aligned CNT sheets drawn from a forest and scrolled into yarn electrodes. The content of MnO_2_ was increased up to 96 wt%, under which an excellent areal capacitance of 3.54 mF/cm^2^ was achieved. Choi et al. [44] also fabricated asymmetric electrode-based yarn supercapacitors. Figure 10b shows the schematic and cross-sectional SEM images of the asymmetric yarn-based supercapacitor prepared using an MnO_2_ and rGO-embedded cathode and anode, respectively. As a result, the operating voltage window was extended to 2 V, i.e., from −1 V to 1 V, and an energy density of 43 μW/cm^2^ was achieved, demonstrating practical availability to operate LEDs. Besides MnO_2_, various materials, including metal oxides, ceramics, and biopolymers, have been used as active materials. MXene, a two-dimensional planar ceramic material, embedded in yarn-based supercapacitors has been reported (Figure 10c) [45].

MXene-dispersed solution-coated CNT sheets were twisted into yarn electrodes, maximizing the loading weight to more than 98 wt%. This high loading weight yields outstanding areal and volumetric capacitances of 3188 mF/cm^2^ and 1083 F/cm^3^, respectively. In addition, embedded yarn-based supercapacitors with RuO_2_ anodes and MXene cathodes woven into a textile powered an LED (for more than 60 s), commercialized electronic devices such as electronic watches, and a digital timer. J. Ren et al. [46] fabricated ordered mesoporous carbon (OMC) embedded yarn supercapacitor. When 90 wt% of the OMC is loaded, this supercapacitor exhibited linear and areal capacitances of 1.91 mF/cm and 39.7 mF/cm^2^, respectively. Under the same measurement conditions, the capacitances were improved approximately 20 times of MWNT based yarn supercapacitor. Jang et al. [47] added NAD, a biomolecule responsible for cellular energy transduction, to CNT yarn electrodes. The NAD bi-scrolled yarn electrode achieved a loading weight of 85 wt% and an areal capacitance of 55.73 mF/cm^2^, demonstrating the potential for practical applications of supercapacitors in in vivo bio-implantation. Table 2 lists various electrochemical performances of yarn supercapacitors with different electrode structures.

### 3.3. Functionality

Although the primary roles of yarn-based supercapacitors are storing and offering electrical energy, the effort required to realize their additional functionality is a crucial step toward their practical and versatile use. Current research trends predominantly focus on achieving mechanical adaptability and seamless integration with other devices. A key consideration in the development of functionality is mechanical deformability. Yarn-based supercapacitors operate while being worn on the human body; therefore, flexibility and deformability, including stretchability, are required to reliably supply electrical energy while adapting to the complex movements of human joints. Furthermore, concerted efforts toward the integration of yarn-based supercapacitors with other devices are required to expand the functionality of these supercapacitors. The complementary combination of supercapacitors with electronic technologies can diversify the range of wearable electronics. In the subsequent sections, we will introduce the research trends aimed at realizing the mechanical adaptability of these devices and their integration with other devices.

#### 3.3.1. Stretchability

Deformations of a curved body structure or joints involve stretching and bending deformations; therefore, the stretchability of yarn-based supercapacitors must be carefully considered. Stretchable electrodes can be produced in two main ways: (1) using intrinsically stretchable materials or (2) designing geometrically stretchable structures that can change shapes. Previously reported yarn supercapacitors were composed of flexible, nonelastic materials that respond to bending but not stretching deformations. Therefore, structural approaches involving coiling, buckling, and hybrid structures are being actively researched.

**Figure 11:**
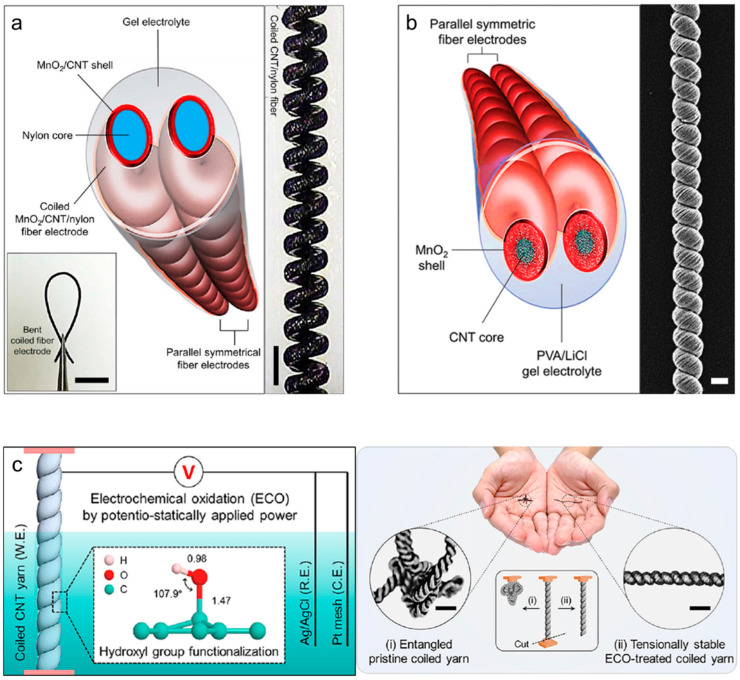
(**a**) Schematic showing a complete all-solid-state supercapacitor, which comprises two symmetric coiled MnO_2_/CNT/nylon fiber electrodes and a gel electrolyte. Optical photographs showing bending and holding with tweezers (scale bar = 5 mm) and a stretched, coiled MnO_2_/CNT/nylon fiber electrode (scale bar = 300 μm). Reproduced under the terms of the CC BY 4.0 license [48]. (**b**) Schematic showing a complete solid-state coiled supercapacitor, which comprises two symmetric MnO_2_/CNT core–shell coiled electrodes, and an SEM image of the MnO_2_/CNT coiled electrode (scale bar = 50 µm). Reproduced with permission [49]. Copyright 2016, Wiley-VCH. (**c**) Schematics showing the experimental setup for electrochemical oxidation and the molecular structure of the functionalized hydroxyl groups in the C=C bond sp2 network. Photographs of (i) entangled pristine coiled yarn and (ii) tensionally stable coiled yarn placed on both palms and optical images of their magnifications (scale bars = 300 μm). Inset shows the morphological changes in the (i) pristine and (ii) stable coiled CNT yarns when one end of each yarn is cut. Reprinted with permission [51]. Copyright 2022, American Chemical Society.

Coiled structures are often found in natural and artificial objects (e.g., plant vines, telephone wire, and automobile suspension systems). These structures are the most widely used structures for fabricating stretchable yarn devices, as the efficient large-scale fabrication of stretchable yarn electrodes is possible. When one end of a yarn is continuously twisted, a spring-like coil loop is gradually formed as mechanical stress is structurally absorbed. Coiled structures highly compress and store yarn lengths in the coil loops and enable stretching in the lengthwise direction under mechanical deformation, leading to structural elasticity being imparted to a nonelastic material. Recently, coil structures have been widely applied in diverse research fields, including stretchable electrodes, energy storage devices, energy harvesters, and actuators. Choi et al. [48] fabricated a coil-structured yarn-based supercapacitor using aligned CNT sheets as the current collector and MnO_2_ as the active material. Figure 11a shows the schematic and an optical image of the all-solid-state coiled yarn supercapacitor composed of MnO_2_, CNT, and nylon. The coiled yarn was fabricated by inserting twists into the nylon core fiber wrapped in aligned CNT sheets and containing pseudocapacitive MnO_2_. The opening of the coiled loop yielded a reversible stretchability of 150%, while the linear compression effect of coiling yielded maximum linear and areal capacitances of 5.4 mF/cm and 40.9 mF/cm^2^, respectively. Figure 11b shows the MnO_2_/CNT-based stretchable coiled yarn-based supercapacitor [49]. The CNT coiled yarn was prepared by applying huge twists (50,000 times per meter) to align the CNT sheets, and then MnO_2_ was electrochemically deposited. The porosities of the aligned CNTs allowed MnO_2_ to effectively infiltrate the interior of the yarn, resulting in a reasonable volumetric capacitance of 34.6 F/cm^3^ (based on a single electrode) and maximum stretchability of 37.5% in the lengthwise direction. Furthermore, an embedded-structure-based coiled yarn supercapacitor with considerably high electrochemical capacitance has been reported [50]. The coiled yarn was created by inserting additional twists into MnO_2_/CNT yarn electrodes produced via bi-scrolling, leading to large areal and volumetric capacitances (382.2 mF/cm^2^ and 104.7 F/cm^3^, respectively) and a mechanical stretchability of 30%. Meanwhile, our research group [51] improved the electrochemical capacitance without adding external active materials and by activating individual CNTs only. As shown in Figure 11c, CNT nanobundles were electrochemically oxidized by applying a potentiostatic voltage to the CNT coiled yarn containing three-electrode electrochemical cells. Although several oxygen-containing functional groups were formed on individual CNT nanobundles, the coiled yarn shape was maintained, exhibiting reversible stretchability of up to 80% with tensional and torsional stability under nontethered states. Moreover, the pseudocapacitive effects of the oxygen-based functional groups in the oxidized CNT yarn increased by approximately 17 times with respect to pristine CNT coiled yarn (linear and areal capacitances of 12.48 mF/cm and 172.93 mF/cm^2^, respectively).

**Figure 12:**
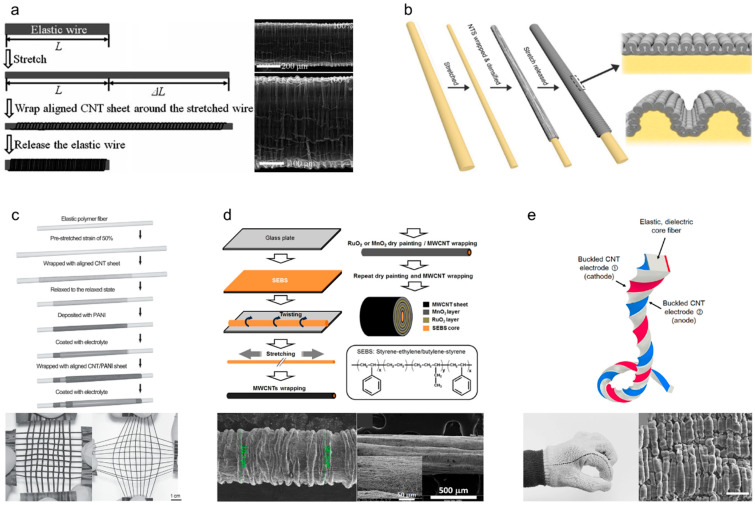
(**a**) Schematic of the fabrication of stretchable conducting wires by wrapping aligned CNT sheets around a pre-stretched elastic wire, and SEM images showing buckled CNTs wrapped on the elastic wire with 100% pre-strain. Reproduced with permission [52]. Copyright 2014, Wiley-VCH. (**b**) Schematic showing the fabrication procedure used to produce MWNT-wrapped CNT yarn and the structural difference between the presently observed buckles for downsized superelastic conducting fibers and the hierarchical buckles observed in previously described millimeter-diameter superelastic conducting fibers. Reproduced with permission [53]. Copyright 2016, Wiley-VCH. (**c**) Schematic showing the fabrication of a superelastic fiber supercapacitor, and optical photographs showing the flexible and stretchable textile before and after stretching by 100%. Reproduced with permission [54]. Copyright 2014, Wiley-VCH. (**d**) Schematic showing the fabrication process for and structure of a stretchable MWNT-based fiber pseudocapacitor. Magnified SEM images showing the surface of the stretchable pseudocapacitor before and after 200% stretching. Reproduced under the terms of the CC BY 4.0 license [55]. (**e**) Schematic showing the twist-inserted rectangular sandwich fiber, which comprises an Ecoflex rubber core and two symmetric, buckled CNT electrodes. Optical photographs showing the 20 cm long sandwich fiber woven into a commercial glove, and an SEM image showing the microscopic CNT buckles (scale bar = 50 μm). Reproduced with permission [57]. Copyright 2023, American Chemical Society.

Buckling (also known as wrinkling) is another effective structural approach for achieving stretchability. When a nonelastic material (e.g., CNTs, graphene, or metal nanowires) is loaded onto a pre-stretched elastic substrate, a stress difference between the nonelastic and pre-stretched layers (or a strain mismatch) is generated, resulting in surface buckling structures. Various morphological characteristics, such as the wavelength and height, of the surface buckles depend on the thickness, Young’s modulus, and Poisson’s ratio of each layer, as well as on the pre-strain. Unlike coiled structures, which are structurally unstable due to the complicated mechanical strain, including tensile and shear strains, buckled structures are stable and stretchable because only tensile strain is applied during fabrication. As shown in Figure 12a, Chen et al. [52] reported a 350% stretchable buckled yarn electrode fabricated by wrapping aligned CNT sheets on pre-stretched elastic polymer wires. Then, poly(3,4-ethylenedioxythiophene)-poly(styrenesulfonate) (PEODT:PSS) was coated on the CNT buckles to store electrical energy using the pseudocapacitance effect. This buckled yarn supercapacitor exhibited an excellent specific capacitance of 30.7 F/g and a retention performance of 97% even at 350% strain. Wang et al. [53] reported newly developed buckling structures called hierarchical buckling structures, in which microbuckles are formed within macrobuckles. As shown in Figure 12b, a hierarchically buckled yarn electrode was fabricated by considerably increasing the amount of CNTs loaded on pre-stretched elastic core fibers, thereby maximizing the strain mismatch. As a result, morphological changes in the buckles were observed depending on the loading amounts of the CNTs, and a stretchability of 1000% originating from the hierarchical buckling structure showed the possibility of fabricating an ultra-stretchable yarn-based supercapacitor. Therefore, a specific capacitance of 79.4 F/g was maintained during 300% stretching deformation. Zhang et al. [54] confirmed the scalability of stretchable yarn-based supercapacitors to energy-supplying textiles. A coaxial CNT/PANI stretchable yarn-based supercapacitor was prepared by repeatedly wrapping aligned CNT sheets and depositing PANI on pre-stretched polymer fibers (Figure 12c). As a result, areal and specific capacitances of 50.1 mF/cm^2^ and 111.6 F/g, respectively, were achieved. Subsequently, 18 strands of stretchable yarn-based supercapacitors were woven into a plain-weave textile by crossing each other, i.e., nine strands are wefts and the other nine strands are wrapped. The textile was found to retain its electrochemical capacitance under 200% biaxial stretching deformation. Figure 12d shows a cable-type stretchable yarn-based supercapacitor with concentric multilayers [55]. RuO_2_ and MnO_2_ were alternately coated onto the MWNT layers wrapped on a pre-stretched SEBS core fiber. The surface buckles allowed the cable supercapacitor to mechanically stretch up to 200% while maintaining a specific capacitance of 25 F/g. Zhao et al. [56] fabricated a high-performance stretchable 1D supercapacitor by winding intrinsically stretchable AuNW/Au film/PANI electrodes in a double helical configuration. By applying the prestrain of 300% and releasing, the wrinkles and cracks were induced by the dimensional changes in fiber. In summary, the resulting 1D stretchable supercapacitors exhibited an impressive specific capacitance of 16.8 mF/cm^2^, and their capacitive performance remained highly stable even when subjected to tensile strains of up to 360%. Meanwhile, a stretchable fiber supercapacitor that integrates all the electrochemical components into one body using a unique design for the yarn structure has been reported. Figure 12e shows a sandwich-structured fiber supercapacitor in which buckled CNT electrodes were placed parallel on both sides of a dielectric polymer rod [57]. Each electrode was coated with MnO_2_ via electrochemical deposition, and both high linear and areal capacitances of 2.38 mF/cm and 11.88 mF/cm^2^ were achieved, respectively. This unique structure maintains the electrochemical capacitances at up to 95% under various mechanical deformations, such as stretching, twisting, and bending, without generating electrical short circuits.

**Figure 13:**
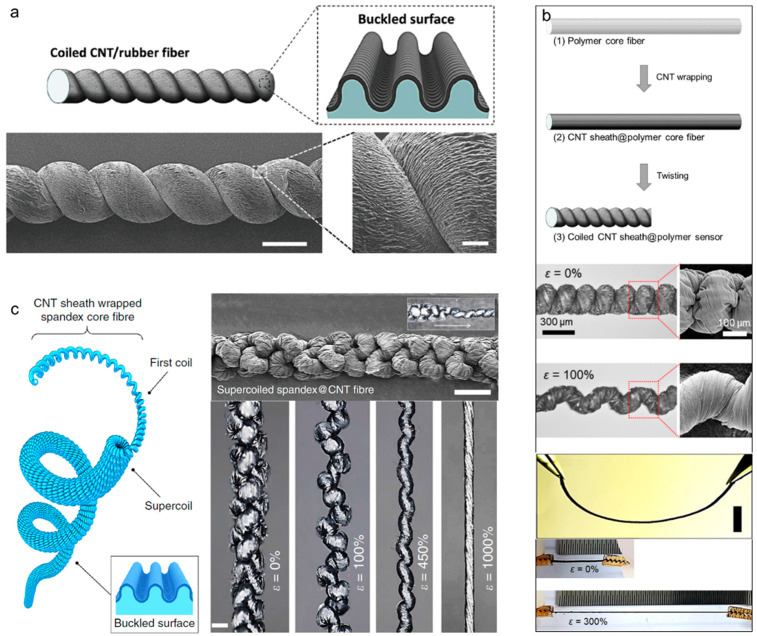
(**a**) Schematics and SEM images showing the CNT-wrapped coiled fiber and magnified buckled surface (scale bar = 500 µm). Reproduced with permission [58]. Copyright 2016, Wiley-VCH. (**b**) Schematics showing the fabrication procedures for coil-structured CNT/polymer fibers, optical photographs, and magnified SEM images showing progressive coil-loop opening from 0 to 100%. Photographs of the bent, pristine, and stretched coiled CNT/polymer fiber (initial length: 1 cm and final length: 4 cm). Reproduced under the terms of the CC BY 4.0 license [59]. (**c**) Schematic showing the highly twisted CNT-wrapped spandex fiber consisting of the first coil, supercoils, and a buckled surface. SEM images showing the supercoiled yarn, fiber transformations from the first coil to the supercoil (scale bar = 200 μm), and optical images of the supercoiled fiber showing progressive coil-opening during application of 0, 100, 450, and 1000% strains (scale bar = 200 µm). Reproduced under the terms of the CC BY 4.0 license [61].

In addition to the aforementioned stretchable yarn structures, hybrid structures that combine coils, buckles, or ply and high-order coiled structures involving coils of coiled yarn have been reported. Figure 13a,b show stretchable fibers in which coiled and buckled structures coexist in one fiber electrode. Choi et al. [58] fabricated microscopically buckled and macroscopically coiled fiber electrodes by inserting twists into buckled fibers prepared via the strain mismatch between the core elastic Ecoflex fiber and surface-wrapped CNT sheet. Linear compression of the coil enabled linear and areal capacitances of 4.8 mF/cm and 22.8 mF/cm^2^, respectively, and the synergistic effect of the coiled and buckled structures imparted outstanding capacitance retention performances of up to 90% under a tensile strain of 800%. Our research group employed commercially available spandex fibers to fabricate the coiled and buckled hybrid fiber structures [59]. As a result, the prepared fiber supercapacitor could be applied to both fiber-type strain sensors and stretchable fiber supercapacitors, resulting in linear and areal capacitances of 1.12 mF/cm and 11.89 mF/cm^2^, respectively, with a stretchability of 300%. Shang et al. [60] reported double-helix-structured fiber supercapacitors in which two coiled yarns were plied together. After Ppy coating, this supercapacitor exhibited a specific capacitance of 63.6 F/g and an outstanding retention performance of up to 97% during 1000 repeated cycles of tensile deformations at 10 Hz. Our research group successfully fabricated a high-order coiled yarn structure with complex coil loops and reported this structure for the first time (Figure 13c) [61]. This structure was fabricated by inserting enormous twists into buckled CNT yarn electrodes, while the first coiled loop was coiled again to form a supercoil. The supercoil was inspired by the DNA supercoil structure that efficiently stores a large amount of genetic information. During supercoiling, the electrode length was compressed to 15% with respect to the initial yarn length, and linear and areal capacitances of 21.7 mF/cm and 92.7 mF/cm^2^, respectively, with a stretchability of 1000%, were achieved after MnO_2_ deposition. Moreover, hierarchical coiled yarn was fabricated by plying nine strands of buckled CNT yarn electrodes. As a result, linear and areal capacitances of 1.32 mF/cm and 3.74 mF/cm^2^, respectively, and a mechanical stretchability of 800% were demonstrated [62]. Table 3 lists the electrochemical performances of supercapacitors with various stretchable yarn structures.

#### 3.3.2. Functional Integration

Significant advancements in stretchable yarn supercapacitors have enabled the fabrication of fully integrated yarn electronics by incorporating functional electronic devices and power sources within a single fiber. These integrated single-yarn systems hold great significance in the field of yarn electronics. This approach eliminates redundant charging circuits and external electrical connections while reducing the overall size, weight, and cost of the supercapacitors. To achieve integrated yarn electronics, yarn supercapacitors were combined with sensors, solar cells, or nanogenerators. These studies explored the compatibility and synergy of these supercapacitors with other devices, fostering a holistic approach to functionality and applicability.

**Figure 14:**
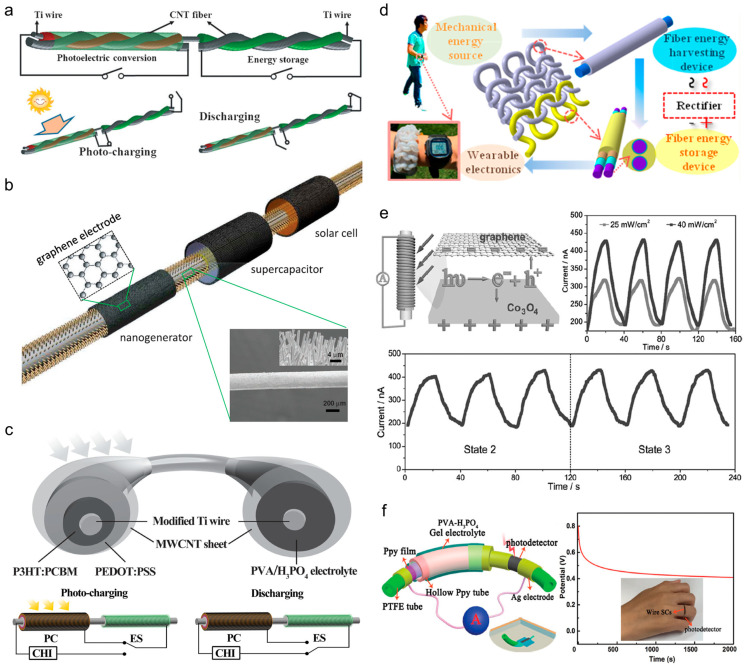
(**a**) Schematics showing the integrated wire-shaped device for photoelectric conversion and energy storage and the circuit connection during the charging and discharging processes. Reproduced with permission [63]. Copyright 2012, Wiley-VCH. (**b**) Self-powered fiber device consisting of a nanogenerator, solar cell, and supercapacitor. Reproduced with permission [64]. Copyright 2011, Wiley-VCH. (**c**) Schematics showing a self-powered fiber with a polymer solar cell and a supercapacitor and the circuit connection state in the charging and discharging processes. Reproduced with permission [65]. Copyright 2014, Wiley-VCH. (**d**) Self-powered textile fabricated by integrating supercapacitors into an energy-harvesting fabric. Reproduced with permission [66]. Copyright 2017, American Chemical Society. (**e**) Schematics showing the integrated system. The current response of the photodetector powered by the flexible asymmetric fiber supercapacitor illuminated under different incident light intensities and at different bending states under one light intensity. Reproduced with permission [68]. Copyright 2014, Wiley-VCH. (**f**) Photo-response characteristics of a flexible integrated-wire supercapacitor-CuInS_2_ photodetection system. Reproduced with permission [69]. Copyright 2018, Wiley-VCH.

As shown in Figure 14a, Chen et al. [63] fabricated an integrated energy wire that combines photoelectric conversion and energy storage functionalities by fabricating dye-sensitized solar cells and supercapacitors on a single yarn. The circuit connections during the charging and discharging processes are shown below. In 2011, Bae et al. [64] reported the first 1D self-powered system. They assembled two energy conversion devices—a nanogenerator and a dye-sensitized solar cell—along with an energy storage component (a 1D supercapacitor) onto a single yarn, employing ZnO nanowires and graphene as active electrode materials (Figure 14b). This configuration allowed for the conversion of mechanical to electrical energy via the nanogenerator and solar energy via the solar cell, subsequently storing this electrical energy within the 1D supercapacitor. Zhang et al. [65] fabricated a flexible fiber containing both a polymer solar cell and a 1D supercapacitor. To construct the solar cell, they initially electrochemically grew Ti nanotubes perpendicular to a Ti wire. Their left regions were then covered with successive layers of poly(3-hexyl thiophene):-phenyl-C61-butyric acid methyl ester, PEDOT:PSS, and finally, an MWCNT sheet. The 1D supercapacitor component was prepared by applying a coating comprised of a PVA/H_3_PO_4_ gel electrolyte layer and CNT sheets wrapped around the right region of the modified Ti wire (Figure 14c). Triboelectric nanogenerators (TENGs) converting mechanical friction into electrical energy can be used as a valuable energy generator for self-powered systems. In 2017, Dong et al. [66] fabricated a self-powered textile by knitting 1D supercapacitors into a TENG fabric and demonstrated that energy can be harvested and stored from human body motions (Figure 14d). Combining yarn supercapacitors with multifunctional sensors to obtain yarn supercapacitor-powered sensing systems represents a crucial and potential pathway for realizing the practical utility of yarn supercapacitors. These integrated systems offer advantages such as reduced weight, exceptional flexibility and stretchability, and seamless integration [67]. Wang et al. [68] presented an asymmetric fiber supercapacitor with Co_3_O_4_ nanowires on Ni fiber (positive electrode) and graphene on carbon fiber (negative electrode), as shown in Figure 14e. Upon application of an external field from the fully charged fiber supercapacitor, the separated electrons moved to the positive electrode (Co_3_O_4_) and the holes to the negative electrode (graphene), thereby increasing the leakage current of the fiber supercapacitor. Thus, photodetection was implemented by monitoring the leakage current changes. Li et al. [69] reported a delicate coaxial fiber supercapacitor in which a CuInS_2_-based photodetector and coaxial supercapacitors were directly constructed on the same polytetrafluoroethylene tube substrate (Figure 14f). Self-charge curves were plotted to determine the stable output voltage. These integrated devices are potentially applicable to wearables.

## 4. Technical Issues

The development of yarn-based supercapacitors for wearables faces several obstacles based on the nature of the materials used and the practicality and safety of these devices for daily use. Carbon-based nanomaterials such as CNTs and graphene are theoretically ideal owing to their flexibility and conductivity, although they are currently too expensive and challenging to mass produce. Nanomaterial production is sensitive to various environmental factors, which makes achieving consistent material properties difficult. Although these supercapacitors perform well in research settings, scaling up the technology degrades their performance. This is mainly due to the increase in electrical resistance with scaling, a problem that is exacerbated by the inherent structural limitations of 1D yarn. Additionally, safety remains a concern in terms of real-world applications. Many of the components used in these supercapacitors, such as metal oxide nanoparticles and acid/base electrolytes, could be hazardous. Another issue is durability, especially when considering the wear and tear associated with regular washing. Finally, for these yarn-based supercapacitors to be widely adopted, they must not only be safe and effective but also comfortable and aesthetically pleasing. The textile industry could contribute to integrating these devices into fabrics that are both functional and fashionable. Each of these challenges presents its own set of research opportunities. Addressing them is, therefore, essential for transitioning yarn-based supercapacitors from the lab to the marketplace.

## 5. Conclusions and Perspectives

Herein, we reviewed the latest advancements and emerging trends in the field of yarn-based supercapacitors. Predominantly, these energy storage devices contain carbon-based nanomaterials such as CNTs and graphene as current collectors and electrodes. To augment the energy storage capabilities, pseudocapacitance effects are induced using metal oxide nanoparticles, such as MnO_2_, RuO_2_, and MoS_2_, along with conductive polymers, such as PEDOT, Ppy, and PANI. Various methods for efficiently loading these active materials, including techniques such as electrodeposition and bi-scrolling, have been developed. Innovative yarn structures that offer features such as stretchability exemplified by the buckle and coil forms have also been introduced.

**Figure 15:**
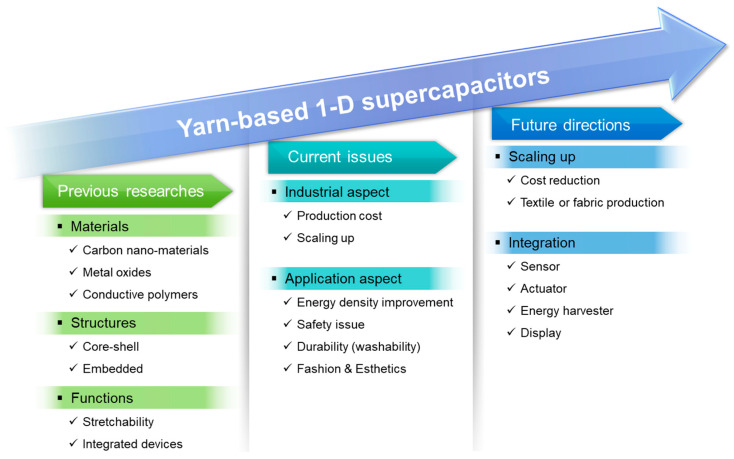
Roadmap of yarn-based 1D supercapacitors, including previous pioneering studies, current research issues, and future research directions.

Pioneering research on yarn-based 1D supercapacitors has made remarkable progress. However, presently, limitations such as high production costs and challenges in terms of mass production, as well as limited energy storage performance, durability, and safety, exist in relation to yarn-based supercapacitor technology [4,70]. To expedite the commercialization of wearable devices, determining future research directions and encouraging collective efforts toward the technological advancement of yarn-based supercapacitors are imperative. The future research directions related to yarn-based supercapacitors have two primary focuses: (1) the transition from laboratory-scale development to industrial-scale production by optimizing nanomaterial synthesis, scaling up production, reducing manufacturing costs, and fabricating textile or fabric forms using yarn-based devices and (2) seamless integration with next-generation energy systems, such as solar cells, fuel cells, and energy harvesters, for real-time energy generation and storage or application in yarn-based devices, such as sensors, conductors, actuators, or advanced electronics, including flexible displays (Figure 15).

These technological advancements offer the potential to operate personalized devices without temporal or spatial constraints, maintaining seamless interconnectivity and being expected to play a pivotal role in achieving hyper-personalization and hyper-connectivity in relation to wearable applications and the Internet of Things. Furthermore, the applications can be extended from wearables and smart clothing to broad sectors such as electric vehicles and robotics. This will enhance the roles of yarn and textiles in technological revolutions. Once at the forefront of the First Industrial Revolution in the 18th century, yarn technology is also crucial to the next industrial revolution three centuries later.

## Figures and Tables

**Table 1:** Comparison of the electrochemical energy storage performances of various materials.

Materials	Ref.	Materials/Electrodes	*C_L_* (mF/cm)	*C_A_* (mF/cm^2^)	*C_V_* (F/cm^3^)	*C_sp_* (F/g)
Carbon Nanomaterials	[22]	CNT fiber/CNT sheet coaxial fiber	0.029	8.66	32.09	59
	[23]	Graphene fiber/3D graphene hybrid fiber	0.02	1.7	-	40
	[24]	Carbon microfiber/MWNT coaxial fiber	6.3	86.8	14.1	11.1
	[25]	rGO-GO-rGO all-in-one fiber	-	2	-	-
	[26]	Wet spinning rGO continuous fiber	-	-	340	279
	[27]	CNT/rGO composite	-	-	68.4	126.7
Metal Oxides	[28]	MnO_2_/MWCNT composite fiber	0.015	3.01	-	13.31
	[29]	MoS_2_/rGO hybrid fiber	-	-	30	-
	[30]	MnO_2_ nanowire/graphene hybrid fiber	-	82.6	66.1	-
	[31]	MnO_2_/GCF (rGO/SWCNT) fiber	3.30	-	-	-
Conductive Polymer	[32]	PEDOT/CNS nanomembrane yarn	-	-	40	21.5
	[33]	PEDOT/MWNT bi-scrolled yarn	-	-	179	-
	[34]	A Ppy/MnO_2_/CNT/cotton thread cable	-	1490	-	-
	[35]	PANI/CNT composite yarn	-	38	-	-
	[36]	PANI/Au/CNT fiber	-	-	-	8.7
	[37]	PAN@PANY/CNT	-	560	-	-

**Table 2:** Comparison of the electrochemical performances of various yarn structures.

Structure	Ref.	Materials/Electrodes	*C_L_* (mF/cm)	*C_A_* (mF/cm^2^)	*C_V_* (F/cm^3^)	*C_sp_* (F/g)
Core–shell	[38]	MnO_2_/G/GF	0.143	9.6	-	36
	[39]	CNT/MnO_2_ composite yarn	-	3.707	25.4	-
	[40]	MnO_2_/carbon fiber	23	-	-	62
	[41]	Single MnO_2_/CFs fiber	-	-	58.7	428
	[42]	Ag/MnO_2_ composite sheath yarn	-	322.2	208.1	-
Embedded	[43]	MnO_2_/CNT fiber	-	3540	-	-
	[44]	rGO/CNT-MnO_2_/CNT fiber	-	322.4	57.2	-
	[45]	MXene/CNT (BMX) yarns	118	3188	1083	428
	[46]	OMC/MWNT fiber	1.91	39.7		
	[47]	NAD/MWNT fiber	-	55.73	-	-

**Table 3:** Comparison of the electrochemical performances of various stretchable yarn structures.

Structure	Ref.	Materials/Electrodes	*C_L_* (mF/cm)	*C_A_* (mF/cm^2^)	*C_V_* (F/cm^3^)	*C_sp_* (F/g)	Stretchability (%)
Coil	[48]	MnO_2_/CNT/nylon coil	5.4	40.9	3.8	-	150
	[49]	MnO_2_/CNT coil	2.72	61.25	34.6	26.5	37.5
	[50]	Bi-scrolled MnO_2_/CNT yarn coil	17.7	382.2	104.7	-	30
	[51]	ECO-treated CNT coil	12.48	172.93	-	-	80
Buckle	[52]	CNT/polymer buckled wire	-	-	-	30.7	350
	[53]	CNT/rubber buckled fiber	-	-	-	~40	300
	[54]	PANI/CNT/rubber buckled fiber	-	50.1	-	111.6	400
	[55]	MnO_2_/RuO_2_/CNT buckled fiber	-	-	-	25	200
	[56]	Au/PANI wrinkled fiber	-	−16.8	-	-	−360
	[57]	MnO_2_/CNT/rubber sandwich	2.38	11.88	-	-	200
Hybrid	[58]	MnO_2_/CNT/rubber buckled coil	4.8	22.8	-	-	800
	[59]	CNT/polymer buckled coil	1.12	11.89	-	-	300
	[60]	Ppy/H_3_PO_4_/CNT plied coil	-	-	-	63.6	150
	[61]	MnO_2_/CNT/spandex supercoil	21.7	92.1	-	-	1000
	[62]	CNT/spandex fiber 9-plied coil	1.32	3.74	-	-	800

## Data Availability

Not applicable.
